# Structure and Implementation of Novel Task Rules: A Cross-Sectional Developmental Study

**DOI:** 10.1177/0956797618755322

**Published:** 2018-05-10

**Authors:** Frederick Verbruggen, Rossy McLaren, Maayan Pereg, Nachshon Meiran

**Affiliations:** 1 Department of Experimental Psychology, Ghent University; 2 School of Psychology, University of Exeter; 3 Department of Psychology, Ben-Gurion University of the Negev

**Keywords:** cognitive development, rule implementation, task instructions, intention-based reflexivity, interference, open data

## Abstract

Rule-based performance improves remarkably throughout childhood. The present study examined how children and adolescents structured tasks and implemented rules when novel task instructions were presented in a child-friendly version of a novel instruction-learning paradigm. Each miniblock started with the presentation of new stimulus-response mappings for a go task. Before this mapping could be implemented, subjects had to make responses in order to advance through screens during a preparatory (“*next”*) phase. Children (4–11 years old) and late adolescents (17–19 years old) responded more slowly during the *next* phase when the *next* response was incompatible with the instructed stimulus-response mapping. This instruction-based interference effect was more pronounced in young children than in older children. We argue that these findings are most consistent with age-related differences in rule structuring. We discuss the implications of our findings for theories of rule-based performance, instruction-based learning, and development.

People often have to perform novel tasks or actions. The present study examined two critical aspects of novel task performance, namely the abilities to follow instructions and to structure tasks hierarchically. These two issues are related when novel task instructions have to be deferred. For example, when you are about to travel to the United Kingdom for the first time, a friend may tell you that you have to look to the left when crossing a street. However, you should follow her instructions only once you have reached your destination, and failure to do so could have serious negative consequences. Here, we tested how children and late adolescents performed in such novel task situations.

## From Instructions to Rule-Based Behavior

When instructions are presented, a task “model” or “set” has to be created. This involves selecting and gating information from the perceptual and motor systems (Cole, Laurent, & Stocco, 2013) and chunking relevant task components (Bhandari & Duncan, 2014). Such cognitive structures allow flexible and rule-based behavior in complex environments (Bhandari, Badre, & Frank, 2017).

Once task structures are created, they have to be implemented. Instructed rules have powerful effects on behavior when they are implemented or maintained for future use (Meiran, Liefooghe, & De Houwer, 2017). Indeed, even if their execution is deferred (as in the example above), rules can influence ongoing performance. In a recent study, subjects were presented with novel instructions at the beginning of each miniblock (Meiran, Pereg, Kessler, Cole, & Braver, 2015a). These instructions described the stimulus-response mapping for the go phase of the block (e.g., “© = left, £ = right”). Before subjects could apply these instructions, they had to advance through a *next* phase (the go and *next* phases were indicated by the stimulus color). In this phase, stimuli were presented, but their identity could be ignored, and subjects simply had to press the same *next* key on each trial (which was either the left or right key). Even though the stimulus-response rules had never been applied before, subjects were slower to respond to *next* stimuli when the *next* response and the go response were incompatible (“£” requiring a left response in the *next* phase but a right response in the go phase) compared with when they were compatible (“©” requiring a left response in both phases). This instruction-based interference effect shows that instructions enable “automatic” task performance (Meiran et al., 2017).

Several lines of research suggest that interference during the task-implementation or execution phases can be reduced by creating hierarchical task structures (Cole, Braver, & Meiran, 2017). In a hierarchical task structure, a task cue (such as stimulus color) or context determines the relevant response rules. Such hierarchical information can shield ongoing tasks (e.g., traveling to the airport) from pending instructions (e.g., walking in London), thereby reducing instruction- or rule-based interference.

## The Development of Structuring and Implementing Rules

Rule-based behavior improves remarkably from infancy through childhood and adolescence (Bunge & Crone, 2009; Diamond, 2013). Such developmental improvements might be due to the ability to create and use hierarchical task structures (Bunge & Zelazo, 2006). For example, Amso, Haas, McShane, and Badre (2014) manipulated hierarchical structure (number of subtasks or branches) and number of competing alternatives within a branch independently. Age-related performance differences were primarily influenced by task structure rather than competition between choice alternatives (see also Unger, Ackerman, Chatham, Amso, & Badre, 2016). In other words, the ability to structure rules improved throughout childhood.

Other studies also found age-related differences in the implementation phase. For example, Zelazo, Frye, and Rapus (1996) observed a dissociation between knowing and doing in 3-year-olds. In a simple rule-switching paradigm, 3-year-olds kept doing the task they started with, even when instructed to perform the other task instead. Importantly, when the children were asked what the task rules were, they could accurately recall them, suggesting they experienced difficulties with implementing (but not remembering) the appropriate rules. The *proactive-control* literature also suggests that young children are less likely to implement or maintain rules than older children (Munakata, Snyder, & Chatham, 2012). This could be due to increased costs associated with advanced rule implementation. For example, Blackwell and Munakata (2014) showed that adding a secondary task to a card-sorting task particularly impaired performance of young children who tried to maintain task-related information over time (compared with children who did not maintain the rules). Thus, for young children, implementing rules in advance comes with challenges and can produce behavioral costs.

## The Present Study

To date, most developmental studies have focused on rule-based performance in situations in which children alternated between well-practiced tasks. This research largely ignores the early stages in which the novel instructions are presented and implemented for the first time (i.e., the first trials or blocks are usually practice and not further analyzed). However, task structures created in the beginning of the experiment determine future task performance (Bhandari & Duncan, 2014). In other words, these early phases are crucial.

**Fig. 1.**
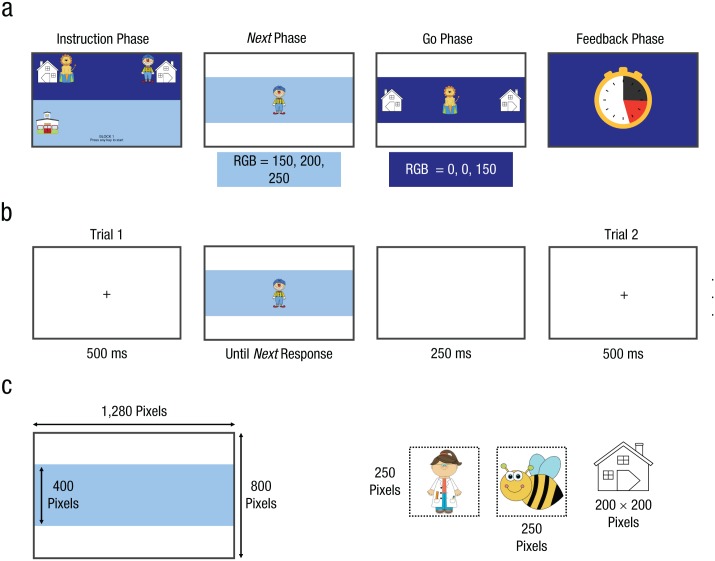
Experimental design. The top two rows show the four phases of each miniblock (a) and the trial course for *next* trials (b). The trial course for go trials was very similar to the course for *next* trials, except that the stimulus disappeared as soon as a response key (correct or incorrect) was pressed. Red, green, and blue (RGB) values are given in (a) for the two background colors. The size of the screen and the stimuli are shown in (c).

The present study examined age-related differences in the task-formation and early implementation stages when novel task instructions were presented. We developed a child-friendly version of the *next* paradigm (Meiran et al., 2015a). This task combines two elements that are usually studied separately, namely the ability to follow or implement instructions and the use of hierarchical structures to shield pending instructions. At the beginning of each miniblock, we showed the children two cartoon images of their “friends” (task-instruction phase; Fig. 1). New images were used for each miniblock. Some of their friends lived on the left side of the street, and some of them lived on the right side. In the evening (go phase), they had to bring their friends home by pressing the appropriate left or right key (task-implementation phase). However, in the morning, before they could go home, all friends had to go to school first (*next* phase), which was located on the left side of the screen for half of the subjects and the right side for the other half. The go and *next* phase were indicated by morning and evening screen backgrounds, respectively. Children (4–11 years old) and late adolescents (17–19 years old) performed this task.

Hierarchical control is needed in this task, since the *next* and go phases create two different contexts. As discussed above, the ability to contextualize behavior and structure tasks develops in young childhood. This ability would reduce interference from one context (go) to the other (*next*). Therefore, the *hierarchical-structure* account predicts that instruction-based interference effects (i.e., slower responding when the *next* response is incompatible with the instructed go response) should be more pronounced for younger children than for older children.

Task rules have to be implemented or maintained in a highly accessible state for an instruction-based interference effect to be observed (Meiran et al., 2017). Theoretical analyses link automatic effects of instructions to proactive control (Cole et al., 2017), but as noted above, young children are less likely to implement rules in advance. Therefore, the *advance-implementation* account predicts impaired performance in the go phase, but less pronounced interference effects in the *next* phase for younger children than for older children (contrasting with practice-based interference effects that are typically larger for younger children; e.g., Huizinga, Dolan, & van der Molen, 2006).

## Method

### Subjects

One hundred seventy-eight children (4–11 years old) from two local schools in Devon (United Kingdom) and 30 late adolescents (17–19 years old) from two local colleges (also in Devon) participated in this experiment (Table 1). We excluded 5 children because they did not complete the experiment and 7 children because accuracy in the go phase was below 60%. In the Supplemental Material available online, we show that excluding these subjects or certain trial types (see below) did not alter the main findings.

**Table 1.** Number of Subjects and Gender for Each Age Group

Age group	*N*	Number of females
4-year-olds	8	6
5-year-olds	22	12
6-year-olds	26	13
7-year-olds	20	11
8-year-olds	29	17
9-year-olds	30	8
10-year-olds	15	6
11-year-olds	16	9
17- to 19-year-olds	30	16

We aimed to recruit as many children and adolescents as possible. Therefore, we contacted two local primary schools, and all children for whom we obtained parental consent were invited to participate. Because we did not know in advance how many parental consent forms we would obtain, we could not determine the exact target sample prior to the experiment. The decision to stop testing was not influenced by the analyses of the data.

The children received a small prize (a sticker of a cartoon character of their choice and a certificate). The adolescents received monetary compensation (£2.50). The experiment was approved by the local research ethics committee. For the children and underage adolescents, parental informed consent and the subjects’ assent were obtained. We obtained written informed consent from the other adolescents.

### Procedure

The experiment took place in a quiet room at school (the children) or college (the adolescents) and was run on a 13-in. MacBook Pro using the Psychophysics Toolbox (Brainard, 1997). We tested one subject at a time. Stimuli consisted of cartoon images of various animals, imaginary creatures, and people. We used different stimuli in each miniblock, and they were easily distinguishable from each other. The “a” and “l” keys of the keyboard were the response keys, and we put arrow stickers on them as a reminder. Both keys were used in the go phase. For half of the subjects, the “a” (left) key was the *next* response; for the others, the “l” (right) key was the *next* response.

Each miniblock consisted of four phases: an instruction phase, a *next* phase, a go phase, and a feedback phase (Fig. 1a). In the instruction phase, we presented the novel stimulus-response mappings for the go phase, and a response reminder for the *next* phase (i.e., a school building on the left or right of the screen, depending on the counterbalancing of the *next* response). The go information appeared on the top of the screen against a dark-blue background (“evening”); the *next* reminder appeared on the bottom against a light-blue background (“morning”). The instructions remained on the screen until subjects had pressed a key and at least 3 s had elapsed.

The trial course of the *next* phase, indicated by a light-blue background, is depicted in Figure 1b. After an intertrial and fixation interval, a stimulus appeared and remained on screen until the correct *next* key was pressed. Thus, if subjects pressed the incorrect key first (e.g., “l” when the *next* response was “a”), the stimulus would remain on the screen; it would only disappear once they had pressed the *next* key. The number of *next* trials differed between blocks (see below). The go phase, indicated by a dark-blue rectangle, always consisted of two trials. The trial course was the same as in the *next* phase, except that the stimulus disappeared as soon as a response key (correct or incorrect) was pressed.

In the feedback phase, we presented a “clock” (Fig. 1a). A dark-gray area on the clock face depicted the total response latency for the two go trials. For each incorrect go response, we added a time penalty (indicated by a red area on the clock face). We also played a sound during the feedback phase: If subjects did not make go errors, we presented the sound “yihaa” (if they had responded faster than in the preceding miniblock) or “ok” (if they had responded slower); we presented the sound “oops” if they had made a go error. The feedback remained on the screen for 1.5 s, after which the following miniblock started.

The experiment consisted of a practice phase and an experimental phase. The practice phase consisted of two parts. First, we explained the main task (see Fig. S1 in the Supplemental Material for the main instructions), and subjects could practice the *next* and go responses. Then we presented three miniblocks that consisted of the instruction, *next*, go, and feedback phases. The practice miniblocks consisted of zero, one, or two *next* trials (each number of *next* trials occurred once, and the order was randomized).

The experimental phase consisted of 48 miniblocks. Twenty-four miniblocks consisted of one *next* trial, 16 consisted of two *next* trials, and 4 consisted of three *next* trials; in 4 miniblocks, the go phase started immediately (so there were no *next* trials). We used this trial distribution to make the start of the go phase unpredictable and to encourage preparation. The order of the miniblocks was further pseudorandomized: Two of the first 10 miniblocks were zero-*next* blocks. Again, this was done to encourage preparation. Subjects received a break after every 12 miniblocks; they could determine the duration of the break themselves. The whole experiment lasted 10 to 15 min (although the youngest children sometimes took a little longer).

### Dependent variables and analyses

All data processing and analyses were completed using R software (R Core Team, 2016). Anonymized data files, R scripts, and experiment documentation are available on the Open Science Framework (https://osf.io/am4yk/).

For the *next* analyses, we focused on the first *next* (*Next* 1) trial because the instruction-based interference effect is largest on the first trial (Meiran et al., 2015a), and performance on later *next* trials could already be modulated by stimulus-specific practice effects. We decided on this before data collection had started. We excluded miniblocks in which subjects made go errors, as these could indicate that subjects did not process the instructions (resulting in a data loss of 17%). We focused on three dependent variables. First, we analyzed the probability of a correct *Next* 1 trial. Second, we analyzed the latency of the *next* response with all (correct and incorrect) *Next* 1 trials included. This response time (RT) analysis was included in order to make the results comparable with those of Meiran et al. (2015a), who did not examine *next* errors. Furthermore, this measure might be most sensitive, as it combines all trials in which traces of inappropriate motor activity (Everaert, Theeuwes, Liefooghe, & De Houwer, 2014; Meiran, Pereg, Kessler, Cole, & Braver, 2015b) cause interference or, in case the activity is high enough, an incorrect response. Third, we recalculated RTs after exclusion of incorrect *Next* 1 trials. For both RT analyses, we used a trimming procedure: We excluded trials on which RT was less than 100 ms or greater than 10 s; then we calculated the mean and standard deviation, and we excluded RTs that were 2.5 standard deviations above the mean. This trimming was done for each subject and condition separately. This resulted in an additional data loss of 3%. Table 2 shows the average number of trials for each condition and age group.

**Table 2.** Average Number of Trials in the *Next* Analysis for Each Age Group

Age group	Compatible trials	Incompatible trials
4-year-olds	15	14
5-year-olds	18	15
6-year-olds	18	16
7-year-olds	19	16
8-year-olds	18	17
9-year-olds	18	16
10-year-olds	18	16
11-year-olds	19	18
17- to 19-year-olds	20	18

For the go analyses, we focused on two dependent variables: accuracy and RT. For the RTs, we excluded incorrect go trials and used the same trimming procedure as the one used for the *next* analyses (combined, this resulted in a data loss of 15%). For all variables, we analyzed performance using the *ezANOVA* function (Lawrence, 2016) in R with age (in years) as a continuous between-subjects variable and compatibility (the *next* analyses) or trial number (first or second trial in the go analyses) as categorical within-subjects variables. This analysis is very similar to a multiple regression with an interaction term or a standard analysis of covariance (ANCOVA; except that the continuous variable is typically considered a nuisance variable in an ANCOVA, whereas the continuous variable was the main interest in the present study; for a similar approach, see Verbruggen & McLaren, 2017). We performed two sets of analyses. First, we performed the analyses with all subjects included. We grouped all adolescents together and used the same age value for all of them (i.e., 18). Table 3 provides an overview of these analyses. Second, we repeated the analyses without the adolescents in case this “extreme” group had an undue influence on inferential statistics. Table 4 provides an overview of these analyses. Note that the main outcomes of the two sets of analyses were similar.

**Table 3.** Results of the Analyses of Variance Used to Explore the Effect of Age, Compatibility (*Next*), and Trial Number (Go 1 or Go 2) on Performance (All Age Groups Included)

Measure and predictor	Sum-of-squares effect	Sum-of-squares error	*F*(1, 194)	*p*	Generalized η^2^
*Next* accuracy					
Age	0.003	1.439	0.463	.497	.001
Compatibility	0.815	1.372	115.281	**< .001**	.225
Age × Compatibility	0.000	1.372	0.015	.903	.000
*Next* response time (all *next* responses included)					
Age	57,461,009	125,243,644	89.006	**< .001**	.297
Compatibility	3,245,765	10,725,756	58.707	**< .001**	.023
Age × Compatibility	278,590	10,725,756	5.039	**.026**	.002
*Next* response time (correct *next* responses only)					
Age	52,994,412	116,227,799	88.455	**< .001**	.300
Compatibility	1,301,115	7,295,666	34.598	**< .001**	.010
Age × Compatibility	145,125	7,295,666	3.859	.051	.001
Go accuracy					
Age	0.435	2.531	33.334	**< .001**	.138
Trial Number	0.026	0.189	26.967	**< .001**	.010
Age × Trial Number	0.000	0.189	0.116	.734	.000
Go response time					
Age	40,974,659	74,749,414	106.343	**< .001**	.320
Trial Number	16,296,012	12,334,139	256.315	**< .001**	.158
Age × Trial Number	3,960,352	12,334,139	62.291	**< .001**	.043

Note: Age was a continuous numerical variable; thus, the first *df* was 1. Significant *p* values are boldfaced.

**Table 4.** Results of the Analyses of Variance Used to Explore the Effect of Age, Compatibility (*Next*), and Trial Number (Go 1 or Go 2) on Performance (Only 4- to 11-Year-Olds Included)

Measure and predictor	Sum-of-squares effect	Sum-of-squares error	*F*(1, 164)	*p*	Generalized η^2^
*Next* accuracy					
Age	0.042	1.190	5.794	**.017**	.018
Compatibility	0.645	1.120	94.449	**< .001**	.218
Age × Compatibility	0.014	1.120	2.122	.147	.006
*Next* response time (all *next* responses included)					
Age	73,886,993	89,366,724	135.593	**< .001**	.426
Compatibility	2,995,665	10,084,257	48.718	**< .001**	.029
Age × Compatibility	668,873	10,084,257	10.878	**.001**	.007
*Next* response time (correct *next* responses only)					
Age	67,849,107	83,501,518	133.258	**< .001**	.429
Compatibility	1,190,868	6,852,862	28.499	**< .001**	.013
Age × Compatibility	419,350	6,852,862	10.036	**.002**	.005
Go accuracy					
Age	0.195	2.309	13.832	**< .001**	.073
Trial Number	0.022	0.169	21.404	**< .001**	.009
Age × Trial Number	0.000	0.169	0.449	.504	< .001
Go response time					
Age	42,987,226	56,421,758	124.950	**< .001**	.393
Trial Number	17,268,650	9,932,973	285.117	**< .001**	.207
Age × Trial Number	4,930,514	9,932,973	81.406	**< .001**	.069

Note: Late adolescents and young adults were excluded from these analyses. Age was a continuous numerical variable; thus, the first *df* was 1. Significant *p* values are boldfaced.

In a pilot study with adults (*N* = 29; see the Supplemental Material), we found medium to large instruction-based interference effects (Cohen’s *d_z_*s = 0.65–1.00). Therefore, we also examined the main effect of compatibility for the different age groups. To increase power and reduce the number of significance tests, we combined the data of the 4- and 5-, 6- and 7-, 8- and 9-, 10- and 11-, and 17- to 19-year-olds, resulting in five groups. Table 5 provides an overview of these analyses.

**Table 5.** Results of Planned Comparisons to Explore the *Next*-Compatibility Effect

Measure and age group	Difference	95% CI	*t*	*p*	Bayes factor	Hedges’s average *g*
*Next-*effect accuracy						
4- and 5-year olds	0.12	[0.072, 0.169]	*t*(29) = 5.042	**< .001**	1,003.94	1.582
6- and 7-year olds	0.073	[0.041, 0.106]	*t*(45) = 4.519	**< .001**	490.01	1.021
8- and 9-year olds	0.105	[0.071, 0.14]	*t*(58) = 6.119	**< .001**	158,677.97	1.361
10- and 11-year olds	0.047	[0.025, 0.068]	*t*(30) = 4.394	**< .001**	205.99	1.402
17- to 19-year olds	0.108	[0.061, 0.155]	*t*(29) = 4.676	**< .001**	397.43	1.571
*Next*-effect response time (all *next* responses included)						
4- and 5-year olds	422	[192, 651]	*t*(29) = 3.761	**.001**	42.13	0.507
6- and 7-year olds	152	[54, 249]	*t*(45) = 3.131	**.003**	10.85	0.272
8- and 9-year olds	162	[99, 224]	*t*(58) = 5.197	**< .001**	6,142.80	0.477
10- and 11-year olds	77	[41, 113]	*t*(30) = 4.362	**< .001**	189.97	0.291
17- to 19-year olds	138	[92, 183]	*t*(29) = 6.174	**< .001**	17,831.86	0.772
*Next*-effect response time (correct *next* responses only)						
4- and 5-year olds	292	[86, 499]	*t*(29) = 2.890	**.007**	5.94	0.356
6- and 7-year olds	105	[29, 182]	*t*(45) = 2.771	**.008**	4.65	0.208
8- and 9-year olds	81	[44, 118]	*t*(58) = 4.389	**< .001**	423.35	0.265
10- and 11-year olds	48	[9, 87]	*t*(30) = 2.529	**.017**	2.86	0.185
17- to 19-year olds	90	[51, 129]	*t*(29) = 4.733	**< .001**	459.03	0.55

Note: Reported *p* values are uncorrected, but all *t* tests were still significant after a Holm-Bonferroni correction. See Schönbrodt and Wagenmakers (2018) for a classification scheme for the interpretation of Bayes factors. We calculated the Bayes factors with the *BayesFactor* package in R using the default prior (0.707). Significant *p* values are boldfaced. CI = confidence interval.

In the main analysis, we focused on the raw RT data. In the Supplemental Material, we report an analysis of proportional instruction-based interference scores. The main numerical trends were similar to those in the analysis reported below.

## Results

### Next phase

**Fig. 2.**
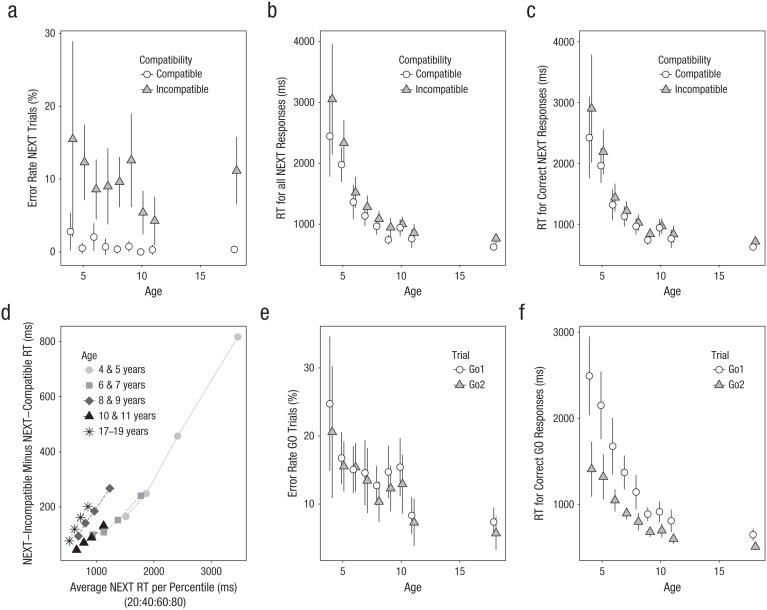
Overview of the *next* and go data (see Method section for a discussion of the different dependent variables). The top row shows mean (a) error rate on *next* trials, (b) response time (RT) for all responses on *next* trials, and (c) RT for correct responses only on *next* trials; results are shown as a function of participants’ age and trial type. The *next*-compatibility effect (d) is shown for the 20th, 40th, 60th, and 80th percentiles; this is for the analysis with all RT trials included. Mean error rate for all go trials (e) and RT for correct go trials only (f) are shown as a function of participants’ age and trial type. Error bars reflect 95% confidence intervals.

We found large interference effects in all analyses: Subjects made more errors and responded more slowly on incompatible trials than on compatible trials (Figs. 2a–2c). This conclusion is supported by the inferential statistics (Tables 3 and 4). Furthermore, the RT analyses revealed general age-related differences. Most importantly, the RT analyses, which included *next* responses that came after erroneously pressing the wrong key, also revealed significant interactions between age and compatibility: The intention-based interference effect decreased over age, which is consistent with the hierarchical-structure account but inconsistent with the advance-implementation account. This decrease can also be seen in Figure 2d, which shows how the intention-based interference effect is influenced by age and overall response speed. For the RT analysis that included only correct *next* responses, the interaction was not significant (*p* = .051) when adolescents were included, but it was significant (*p* = .002) without them (i.e., when the “extreme” group was excluded; see above). The interaction was not significant in both accuracy analyses (*p*s > .14). Table 5 shows that the instruction-based interference effect was significant for all measures and age groups.

### Go phase

The go analyses revealed that error rate and RT decreased over age and that performance was generally worse on the first go trial than on the second go trial. The latter presumably reflects a task-switch cost (for reviews, see Kiesel et al., 2010; Vandierendonck, Liefooghe, & Verbruggen, 2010). The RT cost was larger for the younger children than for the older children and late adolescents, which is consistent with findings reported in the previous literature (Chevalier & Blaye, 2009; Huizinga et al., 2006).

### Exploratory analyses

We also ran an unplanned analysis to explore how the *next* effect evolved throughout the experiment. The stimulus-response mappings changed in every miniblock, so subjects could not practice the mappings. However, they could learn and practice the application of the overall task structure throughout the experiment. Both “fast” and “slow” learning mechanisms could produce such task- or structure-learning effects (Verbruggen, McLaren, & Chambers, 2014). Therefore, we repeated all *next* analyses with experiment half (first 24 miniblocks vs. last 24 miniblocks) as an additional within-subjects variable. Because the number of trials was halved, we had to exclude some extra subjects from the RT analyses because of missing cells after data trimming (1 subject excluded in the *next* all-RT analysis: 6 subjects excluded in the *next* correct-RT analysis).

**Fig. 3.**
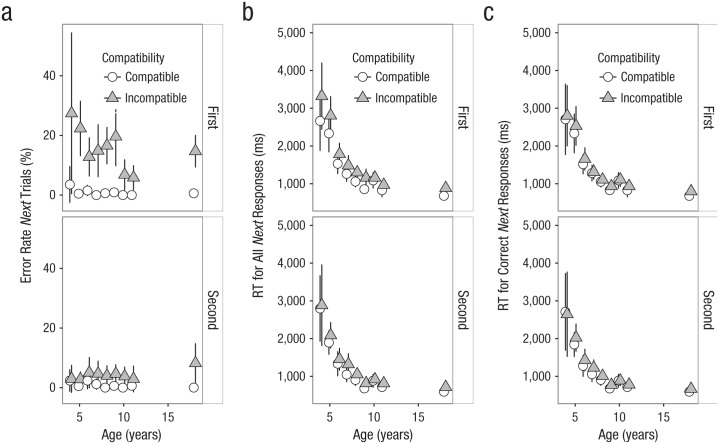
Overview of the *next* data for the first half (top row) and second half (bottom row) of the experiment (see Method section for a discussion of the different dependent variables). The graphs show mean (a) error rate on next trials, (b) response time (RT) for all responses on *next* trials, and (c) RT for correct responses only on *next* trials; results are shown as a function of participants’ age and trial type. Error bars reflect 95% confidence intervals.

The main RT analysis with all *next* responses included (Fig. 3b), revealed that the instruction-based interference effect decreased substantially throughout the experiment (first half: *next* effect = 267 ms; second half: *next* effect = 141 ms; *p* = .007, Table 6). A decrease was observed for all age groups, and the three-way interaction was nonsignificant, *p* = .292. The correct-RT analyses did not reveal any significant interactions between the interference effect and experiment half.

**Table 6.** Results of the Analyses of Variance Used to Explore the Effect of Age, Compatibility, and Experiment Half on *Next* Performance (All Age Groups Included)

Measure and predictor	Sum-of-squares effect	Sum-of-squares error	*F*	*p*	Generalized η^2^
*Next* accuracy					
Age	0.007	2.903	*F*(1, 194) = 0.485	.487	.001
Compatibility	1.723	2.723	*F*(1, 194) = 122.709	**< .001**	.153
Half	0.579	2.101	*F*(1, 194) = 53.420	**< .001**	.057
Age × Compatibility	0.000	2.723	*F*(1, 194) = 0.013	.909	.000
Age × Half	0.044	2.101	*F*(1, 194) = 4.077	**.045**	.005
Compatibility × Half	0.582	1.788	*F*(1, 194) = 63.156	**< .001**	.058
Age × Compatibility × Half	0.059	1.788	*F*(1, 194) = 6.400	**.012**	.006
*Next* response time (all *next* responses included)					
Age	132,682,582	280,640,291	*F*(1, 193) = 91.248	**< .001**	.269
Compatibility	8,124,198	24,107,078	*F*(1, 193) = 65.042	**< .001**	.022
Half	11,404,564	34,832,143	*F*(1, 193) = 63.191	**< .001**	.031
Age × Compatibility	333,362	24,107,078	*F*(1, 193) = 2.669	.104	.001
Age × Half	1,012,000	34,832,143	*F*(1, 193) = 5.607	**.019**	.003
Compatibility × Half	779,212	20,262,113	*F*(1, 193) = 7.422	**.007**	.002
Age × Compatibility × Half	117,153	20,262,113	*F*(1, 193) = 1.116	.292	.000
*Next* response time (correct *next* responses only)					
Age	112,052,500	250,087,632	*F*(1, 188) = 84.234	**< .001**	.267
Compatibility	1,967,512	14,622,138	*F*(1, 188) = 25.297	**< .001**	.006
Half	6,973,384	26,498,290	*F*(1, 188) = 49.475	**< .001**	.022
Age × Compatibility	28,488	14,622,138	*F*(1, 188) = 0.366	.546	.000
Age × Half	634,154	26,498,290	*F*(1, 188) = 4.499	**.035**	.002
Compatibility × Half	188	15,825,461	*F*(1, 188) = 0.002	.962	.000
Age × Compatibility × Half	16,862	15,825,461	*F*(1, 188) = 0.200	.655	.000

Note: Age was a continuous numerical variable; thus, the first *df* was 1. Significant *p* values are boldfaced.

The accuracy analyses also showed that the interference effect decreased during the experiment (Fig. 3a; *p* < .001). Interestingly, significant three-way interactions were observed in the analyses with and without adolescents (Tables 6 and 7). Figure 3 shows that the interference effect decreased more for younger children than for older children. This is consistent with the idea that young children have difficulties with the use of a hierarchical structure but that this improves with some practice. However, it also shows that in the second part of the experiment, the effect was numerically largest for the late adolescents. It seems unlikely that this was due to a floor effect or a speed/accuracy trade-off (e.g., error rates were lower for the 11-year olds than for the late adolescents, yet their *next* RTs were comparable). Instead, this finding could reflect the costs of increased proactive control for the late adolescents. Indeed, go performance was numerically better for the late adolescents. Thus, a possible explanation for these age-related differences is that late adolescents biased the go task to a larger extent than the older children, leading to better go performance but larger costs in the *next* phase. Throughout the experiment, we used the feedback screens to encourage fast and correct go performance, without mentioning *next* performance. This could have induced a go bias and, therefore, higher error rates in the *next* phase. This highlights that proactive control or rule implementation can come with certain costs, even in adolescence.

**Table 7.** Results of the Analyses of Variance Used to Explore the Effect of Age, Compatibility, and Experiment Half on *Next* Performance (Only 4- to 11-Year-Olds Included)

Measure and predictor	Sum-of-squares effect	Sum-of-squares error	*F*	*p*	Generalized η^2^
*Next* accuracy					
Age	0.093	2.407	*F*(1, 164) = 6.327	**.013**	.012
Compatibility	1.36	2.23	*F*(1, 164) = 100.016	**< .001**	.148
Half	0.554	1.747	*F*(1, 164) = 51.981	**< .001**	.066
Age × Compatibility	0.035	2.23	*F*(1, 164) = 2.55	.112	.004
Age × Half	0.061	1.747	*F*(1, 164) = 5.735	**.018**	.008
Compatibility × Half	0.578	1.427	*F*(1, 164) = 66.402	**< .001**	.069
Age × Compatibility × Half	0.062	1.427	*F*(1, 164) = 7.127	**.008**	.008
*Next* response time (all *next* responses included)					
Age	171,404,286	197,300,316	*F*(1, 163) = 141.606	**< .001**	.385
Compatibility	7,358,573	23,003,775	*F*(1, 163) = 52.141	**< .001**	.026
Half	11,360,111	34,047,834	*F*(1, 163) = 54.385	**< .001**	.04
Age × Compatibility	702,690	23,003,775	*F*(1, 163) = 4.979	**.027**	.003
Age × Half	830,297	34,047,834	*F*(1, 163) = 3.975	**.048**	.003
Compatibility × Half	744,969	19,654,374	*F*(1, 163) = 6.178	**.014**	.003
Age × Compatibility × Half	261,020	19,654,374	*F*(1, 163) = 2.165	.143	.001
*Next* response time (correct *next* responses only)					
Age	141,691,900	181,675,437	*F*(1, 158) = 123.227	**< .001**	.374
Compatibility	1,674,591	14,136,173	*F*(1, 158) = 18.717	**< .001**	.007
Half	6,879,870	25,890,490	*F*(1, 158) = 41.985	**< .001**	.028
Age × Compatibility	123,479	14,136,173	*F*(1, 158) = 1.38	.242	.001
Age × Half	622,945	25,890,490	*F*(1, 158) = 3.802	.053	.003
Compatibility × Half	4715	15,657,150	*F*(1, 158) = 0.048	.828	.000
Age × Compatibility × Half	43	15,657,150	*F*(1, 158) = 0	.983	.000

Note: Late adolescents and young adults were excluded from these analyses. Age was a continuous numerical variable; thus, the first *df* was 1. Significant *p* values are boldfaced.

## General Discussion

We examined structuring and implementing novel task instructions in children and late adolescents. We found that subjects’ ability to prepare novel tasks improved with age, as seen in go performance. However, this did not result in an age-related increase in intention-based interference effects: We found interference effects on *Next* 1 trials for all age groups, but these tended to be largest for the youngest children (4- to 5-year-olds).

These results are consistent with the hierarchical-structure account. Situations in which multiple rules can be relevant (in our case, the *next* and go rules) require a hierarchical structure to determine the correct response and to reduce interference between competing task elements. Young children face difficulties with creating or using such structures (Amso et al., 2014; Unger et al., 2016). This could explain the larger instruction-based interference effects for the youngest children. The hierarchical-structure account also receives support from another recent *next* study (Meiran, Pereg, Givon, Danieli, & Shahar, 2016), which demonstrated that adults who were less successful in the go phase, had poorer fluid intelligence, or were generally slower also had a larger *next* effect (i.e., adults with poorer working memory might also experience more problems with hierarchical or complex task sets, somewhat similar to children, than adults with better working memory). Meiran et al.’s (2016) findings are also consistent with research on goal neglect, which suggests associations between fluid intelligence and the ability to chunk task knowledge (Bhandari & Duncan, 2014).

Our results did not provide much support for the advance-implementation account as described in the introduction. Previous developmental work suggests that young children are less likely to implement task rules in advance than older children, adolescents, and young adults. Therefore, the advance-implementation account predicted that go performance would be impaired but the instruction-based interference effect in the *next* phase should be absent (or at least be smaller) for the younger children. Instead, we observed the largest interference effects for the youngest children. The presence of the interference effects and decent go performance indicate that even the youngest children in our sample could implement novel stimulus-response rules in advance. This conclusion is consistent with a study showing that young children engaged in proactive control (i.e., they prepared rules in advance) when the task was more difficult (Chevalier, Martis, Curran, & Munakata, 2015). Here, we used novel stimulus-response mappings in each miniblock. This prevented stimulus-specific practice and the consequent formation of long-term memory traces, which could have encouraged the implementation of the rules during the instruction phase. However, consistent with the results of Blackwell and Munakata (2014), our findings showed that implementing these rules came with a substantial cost in young children (i.e., large interference effects during the *next* phase).

The exploratory analyses revealed that the instruction-based interference effects (in the accuracy and main RT analyses) decreased throughout the experiment. In the accuracy analyses, this effect was most pronounced for the youngest children. The decrease is consistent with findings in adults (Meiran et al., 2015a). In *next* experiments, subjects cannot learn specific stimulus-response associations. However, they may gradually get better at “separating” the go phase (indicated by the dark-blue background) from the *next* phase (indicated by the light-blue background). In other words, we speculate that hierarchical structures (with the context cue modulating the choice options) and their usage further evolved throughout practice, reducing interference between the go and *next* components of the task. This idea is consistent with other findings in the task-learning literature (Bhandari et al., 2017).

By contrasting the hierarchical-structure and advance-implementation accounts, readers may get the incorrect impression that the task-formation and task-implementation phases are independent. But when people create an inefficient nonhierarchical structure or when they have difficulties managing the contingencies within the structure, more competition between the various choice options occurs (producing larger instruction-based interference effects). Thus, task structure will have knock-on effects on the implementation stage. Interestingly, goal neglect (i.e., the dissociation between knowing and doing) has also been associated with the formation of inefficient task structures (Bhandari & Duncan, 2014). This raises the intriguing possibility that failing to implement or execute a task (i.e., goal neglect: a negative “symptom”) and applying the rules when not required (i.e., instruction-based interference: a positive symptom) both arise from a failure to create an efficient task structure. Future research is needed to test how these phenomena are related.

To conclude, we observed intention-based interference effects in all groups, indicating that even the younger children in our sample implemented novel rules at the beginning of each miniblock. We attribute the numerically larger RT costs to age-related differences in the creation of hierarchical task structures. Furthermore, we propose that the *next* paradigm might be a useful tool to study structuring and implementation of instructions in different age groups and, more generally, the powerful effects that instructions and intentions can have on behavior.

## Supplemental Material

VerbruggenFigS1 – Supplemental material for Structure and Implementation of Novel Task Rules: A Cross-Sectional Developmental Study Click here for additional data file. Supplemental material, VerbruggenFigS1 for Structure and Implementation of Novel Task Rules: A Cross-Sectional Developmental Study by Frederick Verbruggen, Rossy McLaren, Maayan Pereg and Nachshon Meiran in Psychological Science

## Supplemental Material

VerbruggenOpenPracticesDisclosure – Supplemental material for Structure and Implementation of Novel Task Rules: A Cross-Sectional Developmental Study Click here for additional data file. Supplemental material, VerbruggenOpenPracticesDisclosure for Structure and Implementation of Novel Task Rules: A Cross-Sectional Developmental Study by Frederick Verbruggen, Rossy McLaren, Maayan Pereg and Nachshon Meiran in Psychological Science

## Supplemental Material

VerbruggenSupplementalMaterial – Supplemental material for Structure and Implementation of Novel Task Rules: A Cross-Sectional Developmental Study Click here for additional data file. Supplemental material, VerbruggenSupplementalMaterial for Structure and Implementation of Novel Task Rules: A Cross-Sectional Developmental Study by Frederick Verbruggen, Rossy McLaren, Maayan Pereg and Nachshon Meiran in Psychological Science
