# Supplementary material for: Structure and Implementation of Novel Task Rules: A Cross-Sectional Developmental Study
Source: Psychol Sci. 2018 May 10;29(7):1113–25. doi: 10.1177/0956797618755322 (PMC6247441; doi:10.1177/0956797618755322)

Appendix: Task instructions.

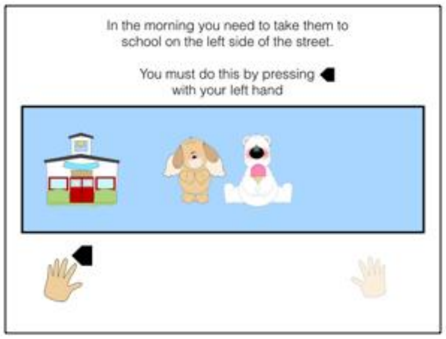

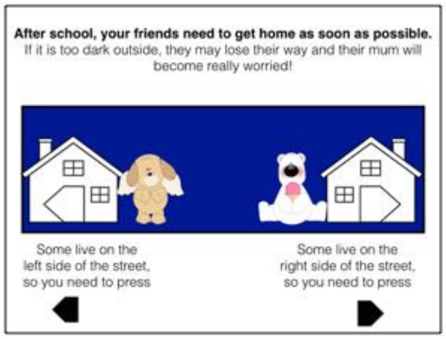

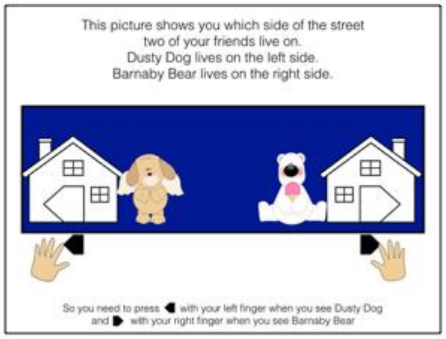

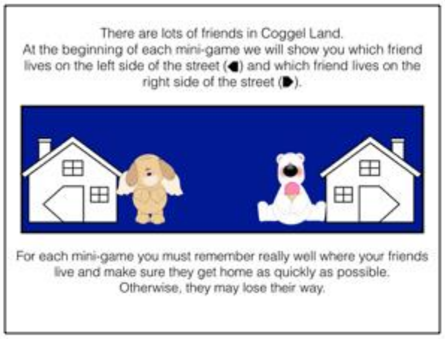

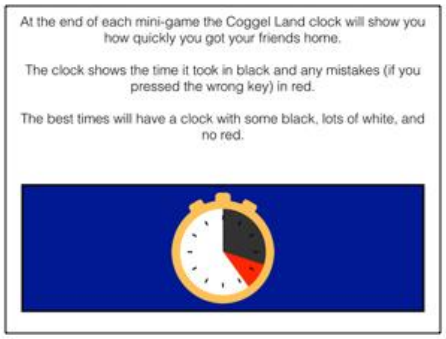

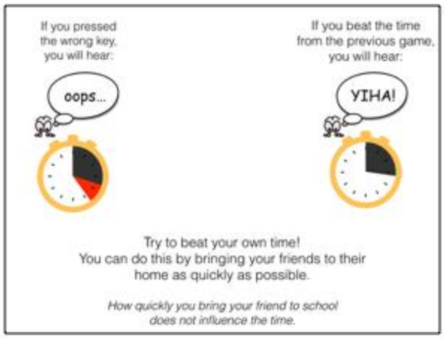

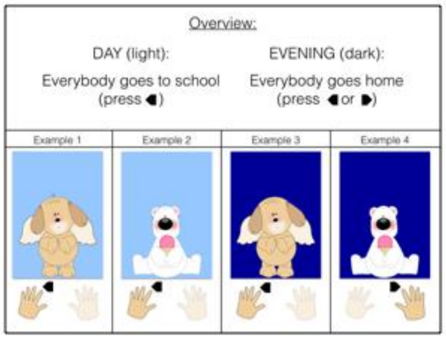

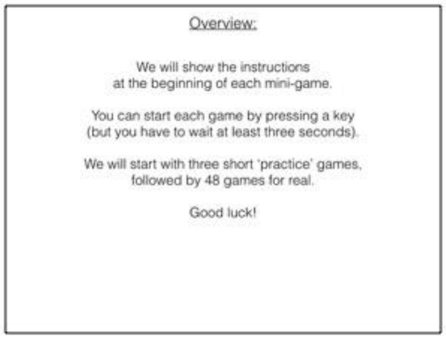

Supplement: VerbruggenFigS1 – Supplemental material for Structure and Implementation of Novel Task Rules: A Cross-Sectional Developmental Study [file VerbruggenFigS1.pdf]
