# Supplementary material for: Structure and Implementation of Novel Task Rules: A Cross-Sectional Developmental Study
Source: Psychol Sci. 2018 May 10;29(7):1113–25. doi: 10.1177/0956797618755322 (PMC6247441; doi:10.1177/0956797618755322)
Supplement: VerbruggenSupplementalMaterial – Supplemental material for Structure and Implementation of Novel Task Rules: A Cross-Sectional Developmental Study [file VerbruggenSupplementalMaterial.pdf]

SUPPLEMENTARY MATERIALS

Analyses with all subjects and miniblocks included

In the analysis reported in the main manuscript, we excluded 5 subjects who did not complete the experiment, and 7 subjects who made more than 40% errors in the GO phase. For the NEXT analyses, we also excluded all miniblocks in which subjects made a GO error. In Table S1, we report the inferential statistics when all subjects and miniblocks are included. As can be seen, the results are very similar to the results reported in the main manuscript.

Table S1: Overview of the ANOVAs used to explore the effect of age, compatibility (NEXT) and trial number (GO1 or GO2) on performance. Age was a continuous numerical variable; thus, df1 = 1. For two subjects (who were excluded from the analyses reported in the main manuscript), we did not have NEXT RT data after the RT trimming.

	Df	*Sum of squares effect*	*Sum of squares error*	*F*	*p*	*generalized η²*
NEXT accuracy						
Age	1,206	0.005	1.440	0.740	.391	0.002
Compatibility	1,206	0.776	1.185	134.925	< .001	0.228
Age x Compatibility	1,206	0.001	1.185	0.177	.675	0.000
NEXT RT (all NEXT included)						
Age	1,204	71731219	152346085	96.052	< .001	0.305
Compatibility	1,204	4083022	10830441	76.907	< .001	0.024
Age x Compatibility	1,204	403846	10830441	7.607	.006	0.002
NEXT RT (correct NEXT only)						
Age	1,204	63278141	133625697	96.604	< .001	0.306
Compatibility	1,204	1541188	9579202	32.821	< .001	0.011
Age x Compatibility	1,204	157680	9579202	3.358	.068	0.001
GO accuracy						
Age	1,206	0.709	3.942	37.070	< .001	0.141
Trial Number	1,206	0.025	0.385	13.405	< .001	0.006
Age x Trial Number	1,206	0.001	0.385	0.295	.588	0.000
GO RT						
Age	1,206	48182914	81816934	121.316	< .001	0.339
Trial Number	1,206	12275878	12055967	209.758	< .001	0.116
Age x Trial Number	1,206	2874675	12055967	49.119	< .001	0.030

Analyses of proportional interference-based scores

We reanalyzed the NEXT RT data using proportional interference-based scores. More specifically, for each subject, we divided mean RT for incompatible NEXT trials by mean RT for compatible NEXT trials. Scores > 1 indicate an interference effect. The descriptive and inferential statistics appear in Tables S2 and S3, respectively.

As can be seen in Table S2, the mean proportional scores were larger than 1 for all age groups. Although the largest value was observed for the 4-year olds, the 95%

confidence interval included 1, which is presumably due to the high variability and low N. Importantly, we again failed to find an increase in instruction-based interference with age, which is inconsistent with the advance-implementation account (see the manuscript).

The raw RT analyses with all NEXT trials included revealed a decrease in the NEXT effect. This decrease was not observed in the proportional analyses. Proportional scores are sometimes used to control for general differences in RT. However, these scores (and other related transformation procedures) should be interpreted with caution as well. Acting or responding to a stimulus involves various cognitive mechanisms or processing steps (e.g. detecting the stimulus, selecting the appropriate response, executing the response; Verbruggen, McLaren, Chambers, 2014). Transforming the data could ‘inflate’ or ‘deflate’ the effect of interest if not all processing stages are affected similarly. For example, if a between-subjects variable (such as age) primarily influences signal detection or motor execution, but the effect of interest is situated at a response-selection level, then using proportional scores will reduce the effect size. In sum, based on the various RT analyses (both with and without incorrect NEXT trials), we cannot confidently conclude that the instruction-based interference effect decreased with age, but we can certainly conclude that it did not *increase* (as predicted by the advance-implementation account).

Table S2: Overview of the descriptive statistics for proportional scores. CI = confidence interval.

Age	NEXT proportional RT (all NEXT trial included)				NEXT proportional RT (only correct NEXT trials)			
	M	SD	lower CI	upper CI	M	SD	lower CI	upper CI
4	1.28	0.36	0.97	1.58	1.22	0.34	0.94	1.51
5	1.18	0.23	1.08	1.28	1.11	0.20	1.02	1.19
6	1.17	0.22	1.08	1.26	1.12	0.14	1.07	1.18
7	1.17	0.35	1.00	1.33	1.11	0.27	0.98	1.23
8	1.16	0.22	1.08	1.25	1.09	0.17	1.02	1.15
9	1.24	0.33	1.12	1.37	1.12	0.14	1.07	1.18
10	1.07	0.09	1.02	1.12	1.04	0.11	0.98	1.10
11	1.14	0.13	1.07	1.21	1.11	0.13	1.04	1.18
17-19	1.23	0.20	1.15	1.30	1.15	0.17	1.09	1.21

SUPPLEMENTARY MATERIALS

Table S3: Overview of the ANOVAs used to explore the effect of age on proportional interference-based scores. Age was a continuous numerical variable; thus, df1 = 1. Consistent with the main analyses, we performed the analyses with and without the adolescents.

	Df	*Sum of squares effect*	*Sum of squares error*	*F*	*p*	*generalized η²*
Adolescents included						
NEXT RT (all NEXT included)	1,194	0.014	12.154	0.231	0.631	0.001
NEXT RT (correct NEXT only)	1,194	0.009	6.400	0.258	0.612	0.001
Only children						
NEXT RT (all NEXT included)	1,164	0.051	10.876	0.773	.381	0.005
NEXT RT (correct NEXT only)	1,164	0.051	5.503	1.528	.218	0.009

Overview of the pilot study

30 undergraduate students from the University of Exeter participated in a pilot study, for partial course credit or monetary compensation (£2.5). One subject was excluded because accuracy in the GO phase was below 60%. The apparatus, stimuli, and procedure were the same as in the main experiment.

The descriptive and inferential statistics appear in Tables S4 and S5, respectively. As can be seen, we observed robust compatibility effects in the NEXT phase, and switch costs in the GO phase. We observed the same pattern of results for all age groups in the main experiment.

Table S4: Overview of the descriptive statistics for the NEXT and GO phases.

	NEXT Error rate		NEXT RT (all trials incl.)		NEX RT (only correct)		GO Error rate		GO RT (only correct)	
	C	IC	C	IC	C	IC	GO1	GO2	GO1	GO2
M	.007	.14	570	728	565	663	.075	.049	569	482
SD	.02	.13	128	176	118	153	.052	.045	80	55

Note: For the NEXT phase, C = compatible, IC = incompatible. For the GO phase, GO1 = the first GO trial, GO2 = the second GO trial.

Table S5: Overview of NEXT (incompatible vs. compatible) and GO (first vs. second GO trials) analyses. Df = 28 for all test. See the main manuscript for a description of the Bayes factors (BF).

	diff	*lower CI*	*upper CI*	*t*	*p*	*BF*	g_{av}
NEXT Error rate	0.13	0.081	0.18	5.407	< .001	2305	1.693
NEXT RT (all NEXT trials included)	158	93	224	4.956	< .001	750	1.027
NEXT RT (correct NEXT trials only)	97	40	155	3.481	.002	21	0.711
GO error rate	0.07	0.011	0.043	3.408	.002	18	0.537
GO RT (correct GO trials only)	87	70	104	10.486	< .001	2.82×10^8	1.271
